# Future Time Perspective and Perceived Social Support: The Mediating Role of Gratitude

**DOI:** 10.3390/ijerph17186707

**Published:** 2020-09-15

**Authors:** Giulia Casu, Eugenia Gentili, Paola Gremigni

**Affiliations:** Department of Psychology, University of Bologna, 40127 Bologna, Italy; eugenia.gentili2@studio.unibo.it (E.G.); paola.gremigni2@unibo.it (P.G.)

**Keywords:** future time perspective, received social support, given social support, dispositional gratitude, mediation analysis

## Abstract

Future time perspective, perceived social support, and dispositional gratitude are topics of interest that positively influence people’s psychological health. Although gratitude has been positively associated with future time perspective and social support, this is the first study to investigate its mediating role in the relationships of future time perspective with perceived social support. A convenience sample of 1256 adults (55.1% women), mean age 34.55 years (SD = 13.92), completed self-reported measures of future time perspective (conceptualized as remaining time and opportunities), dispositional gratitude, and received and given social support. Results indicated that higher dispositional gratitude mediated the relationship between the perception of more remaining time and opportunities and greater perception of received and given social support. These findings highlight the importance of considering dispositional characteristics to better understand the impact of perceived future on the individuals’ subjective experience of social exchanges. Interventions addressing future time perspective and dispositional gratitude might have beneficial effects on individuals’ social well-being.

## 1. Introduction

Time-perspectives, perceived social support, and dispositional gratitude are among the multitude of factors that were found to exert a positive influence on people’s psychological health [1,2,3]. The associations between these dimensions have been separately investigated, showing a positive link of future time perspective with the composition and perceived quality of social relationships [4,5], and a positive link of dispositional gratitude either with future time perspectives or perceived social support [3,6,7]. However, no previous study has simultaneously considered all three dimensions when exploring the link between them; therefore, this study aimed at providing a contribution to fill this gap in the literature. 

Perceived social support refers to the informational, instrumental, emotional and appraisal functions that the exchange activities within one’s social network serve [8]. A distinction has been made between receiving and giving social support [9], which have both been associated with better psychological health outcomes [10,11,12]. Perceived social support is widely treated as an outcome variable in the literature, as it is viewed as an inherent component of social well-being [13]. Studies that explored the relationships of time perspectives or gratitude with social support also used it as an outcome, as we will see just below.

Time perspectives are relatively stable characteristics that express individuals’ preferred attitudes towards the past, present, and future [14]. Although many studies have investigated the effects of all these dimensions, research focused primarily on a single time-domain like the future [1], which we also addressed in the present study. Future time perspective is the individuals’ general tendency to perceive their remaining time in life either as limited or open-ended and expansive [4,15]. Allemand and Hill [6,16], following a multidimensional approach [17], identified remaining time and remaining opportunities as two related but distinct dimensions of future time perspective. For example, a person can see his or her time ahead as expansive and full of opportunities and goals, or as limited but still full of opportunities. A focus on different aspects of the future may differently affect individuals’ priorities, activities, and outcomes [15], including the extension and quality of their social exchanges [4]. The perception that future time is open-ended and full of opportunities was predictive of greater perceived instrumental and emotional support from others [5,18]. Future time perspective was positively related to prosocial behavior and predicted engaging in volunteerism, helping behavior, and giving social support [19]. In summary, perceiving the future as expansive and full of opportunities may lead to reporting a higher level of social support from and given to others. However, few studies have simultaneously considered future time perspective and perceived social support as multidimensional constructs. 

Researchers have conceptualized gratitude on state and trait levels [20]. As an affective-cognitive state, it results from appraising a benefit received from an external source as a positive outcome. As an affective disposition, gratitude is a generalized tendency to be aware of and thankful for the good things that happen [21], and to respond with grateful emotion to the roles of other people’s benevolence [22]. Therefore, it has essential social functions for reciprocal altruism [23]. Empirical evidence showed that higher gratitude led to perceiving more significant social support from others [24], appraising the help received as costly to the benefactor, valuable, and altruistic [25], and engaging in prosocial behavior [3,26]. 

As for the dimensions that may influence gratitude, Szczesniak and Timoszyk-Tomczak [7] found a role for the positive perception of time perspectives. Allemand and Hill [6,16] provided longitudinal evidence that both dimensions of future time perspective (i.e., focus on remaining time and opportunities) were systematically, positively associated with gratitude at the within- and between-person levels. Przepiorka and Sobol-Kwapinska [27] have recently highlighted the influence of positive time perspectives on dispositional gratitude, showing the mediating role of gratitude in the link between time perspectives and positive outcomes, such as life satisfaction. Alternative models with time perspectives as mediators between dispositional gratitude and other dimensions (e.g., well-being) showed no mediating role of future time perspectives [27,28]. In summary, time perspectives were studied as predictors of dispositional gratitude coherently with their conceptualization as primary subjective experiences that may become dispositional characteristics and determine an individual’s choices and actions [29]. 

The present research aimed to investigate whether future time perspectives were associated with perceived social support directly and indirectly, through the mediation of dispositional gratitude. We used a multidimensional approach to future time perspective, intended as remaining time and opportunities [6,16], and social support, intended as received from and given to others [9]. We put forward the following hypotheses. First, since there is evidence of a connection between future time perspectives and perception of social support [5,18,19], we hypothesized a direct association of greater perceived time and opportunities ahead with higher perceptions of received and given social support. Second, since future time perspective predicted dispositional gratitude on the one hand [6,7,16,27], and gratitude was linked to social support on the other hand [3,24,25], we hypothesized that dispositional gratitude would partially mediate the relationship between future time perspective and perceived social support. Specifically, we expected a positive association of remaining time and opportunities with dispositional gratitude and that dispositional gratitude would be positively associated with received and given social support. 

From a practice implication perspective, although future time perspectives are relatively stable over time, various methods have been employed to enable individuals to avoid the negative consequences of an imbalance in their time perspectives [30]. Moreover, if part of the influence of future time perspectives on perceived social support can be attributed to gratitude, supporting our hypotheses, we could intervene to modify this mediator, which has indeed proven to be modifiable with specific training [31]. 

## 2. Materials and Methods 

### 2.1. Participants and Procedure

The present study has a correlational design, and data were collected through convenience sampling. We determined the sample size a priori, as to meet the recommended ratio of 5–10 observations per each estimated parameter in structural equation modelling and to reach enough power (0.80) to detect a mediated effect assuming small-to-medium sizes of the paths [32]. A total of 80 master’s level psychology students attending the course of Psychometrics run by the last author agreed to voluntarily participate in the data collection. Each student was asked to send an e-mail invitation to 20 of his or her family members, friends, or acquaintances, excluding their college friends. Potential participants were invited if they were 18 years or older and able to understand Italian. Students did not fill out the questionnaire as required by the Bioethics Review Board. This convenience sampling strategy was chosen because it required fewer resources than administering the same measures in random samples of the general population. The e-mail invitation briefly described the study and provided a link to an online survey. The first page of the survey contained an informed consent statement that described the study and ensured the participants’ anonymity. Only after clicking the “Yes, I consent to participate” button, respondents were directed to the questionnaires. The University Bioethics Review Board approved the study (Prot. 84196, 8.4.2018).

Of the 1600 individuals invited to participate, 1256 completed the study questionnaires with a 78.5% response rate. Of them, 692 (55.1%) were women and 564 (44.9%) men. The age was between 18 and 80 years (M = 34.55, SD = 13.92). Regarding educational attainment, 655 (52.1%) had up to the secondary school level, and 601 (47.9%) up to the postgraduate level. 

### 2.2. Measures

A socio-demographic section of the survey asked participants about their gender, age, and educational attainment. Questionnaires not available in Italian language were translated and back-translated by two independent bilingual psychologists according to international guidelines for test translation and cross-cultural adaptation [33].

We measured future time perspective using six items from the Future Time Perspective Scale (FTPS) [4], following Allemand and Hill’s studies [6,16]. Three items (e.g., “My future seems infinite to me”) were used to measure the tendency to perceive remaining time as broad and expansive. Three items (e.g., “I feel that many opportunities await me in the future”) were used to assess the tendency to perceive the future as filled with remaining opportunities. Respondents rated each item on a 5-point scale from 1 (*strongly disagree*) to 5 (*strongly agree*). The Italian version obtained for this study was preliminary tested for structural validity and reliability. As evidence of validity, a confirmatory factor analysis (CFA) indicated that the two-factor model proposed by Allemand and Hill [16] fitted the data well (*χ^2^*(8) = 20.97, *p* = 0.01; RMSEA = 0.04; SRMR = 0.02; CFI = 1.00; TLI = 0.99) and significantly better (Δ*χ^2^*(1) = 380.19, *p* < 0.001) than a one-factor model (*χ^2^*(9) = 401.16, *p* = 0.02; RMSEA = 0.04; SRMR = 0.01; CFI = 1.00; TLI = 0.97). Cronbach’s α was 0.70 for remaining time and 0.89 for remaining opportunities, indicating adequate internal consistency.

We measured dispositional gratitude using the validated Italian version [34] of the six-item Gratitude Questionnaire−6 (GQ−6) [22]. Items (e.g., “I have so much in life to be thankful for’’ and “I feel grateful to a wide variety of people”) were rated on a 7-point scale from 1 (*strongly disagree*) to 7 (*strongly agree*). The Italian GQ−6 proved to be a one-factor measure with adequate reliability and expected positive associations with mindfulness and dimensions of psychological well-being [34,35]. In the present study, Cronbach’s α was 0.74.

We measured social support with the 2-Way Social Support Scale (2-Way SSS) [9]. The 21-item 2-Way SSS addresses received (11 items) and given (10 items) emotional and instrumental support (e.g., “When I am feeling down there is someone I can count on”; “If stranded somewhere there is someone who would get me”; “I give others a sense of comfort in times of need”; and “I am a person others turn to for help with their tasks”). Items were rated on a 6-point scale from 0 (*not true at all*) to 5 (*totally true*). In the present research, we used two overall scores for received and given social support, respectively. The validity of using these overall scores in the Italian context was preliminary verified by performing a CFA, which yielded acceptable fit indexes (*χ^2^*(184) = 851.344, *p* < 0.001; RMSEA = 0.08; SRMR = 0.05; CFI = 0.92; TLI = 0.91), according to conventional criteria [36]. As evidence of reliability, Cronbach’s α was 0.93 for received and 0.90 for given social support. 

### 2.3. Data Analysis

Preliminary analyses included descriptive statistics, zero-order correlations among psychological variables, and associations of sociodemographic variables with the mediator and outcomes using Pearson’s correlation coefficient or analysis of variance (ANOVA). A multicollinearity problem was established for correlations > 0.80 among independent variables [37]. Age was included as a covariate in the mediation model if correlated at *r* ≥ |0.30| level; gender and educational attainment were included if significant between-group differences were found on the mediator or outcome scores [38].

We used path analysis to test the direct and indirect (through the mediation of dispositional gratitude) associations of future time perspective dimensions (i.e., remaining time and opportunities) with received and given social support. We evaluated the goodness of fit through the following indexes—root mean square error of approximation (RMSEA) ≤ 0.06, standardized root mean-square residual (SRMR) ≤ 0.08, and comparative fit index (CFI) and Tucker–Lewis index (TLI) ≥ 0.95 [36]. We used a bootstrapping procedure to estimate and test the indirect associations [39]. We performed Chi-square difference (Δ*χ^2^*) tests to compare alternative nested models. 

For interpretation of results, we used both statistical significance (*p* < 0.05 and bootstrapped 95% confidence intervals not including zero for indirect associations) and measures of effect size, with Pearson’s *r* or standardized beta of 0.10 considered small, 0.30 medium and 0.50 large, and Cohen’s *d* of 0.20 considered small, 0.50 medium and 0.80 large [40]. Preliminary analyses were performed using IBM SPSS for Windows version 26 (IBM Corp., Armonk, NY, USA). Path analysis was performed with Mplus 7.4 [41]. 

## 3. Results

### 3.1. Preliminary Results

Descriptive statistics and bivariate correlations between variables are provided in Table 1. Both remaining time and remaining opportunities were positively, moderately associated with dispositional gratitude, and received and given social support. Dispositional gratitude was strong to moderately positively associated with received and given social support. The positive correlation between the remaining time and opportunities was strong (*r* = 0.52) but still does not indicate a multicollinearity problem [37].

Regarding tests for covariates or confounding variables to include in the path analysis, age correlated weakly with the moderator and outcome variables (*r* from −0.12 to −0.10); thus, we excluded it from further analyses. Gender differences in dispositional gratitude were nonsignificant and small. Women reported significantly, slightly greater social support than men, as both received and given. Participants with up to secondary school education reported significantly lower gratitude and received social support than those with up to postgraduate education, whereas differences in given social support were small and nonsignificant (Table 2). Thus, we included gender and educational attainment as covariates in the mediation model.

### 3.2. The Mediation Model

**Figure 1:**
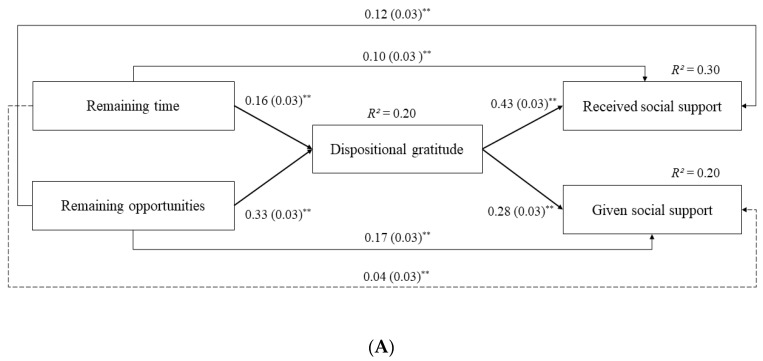

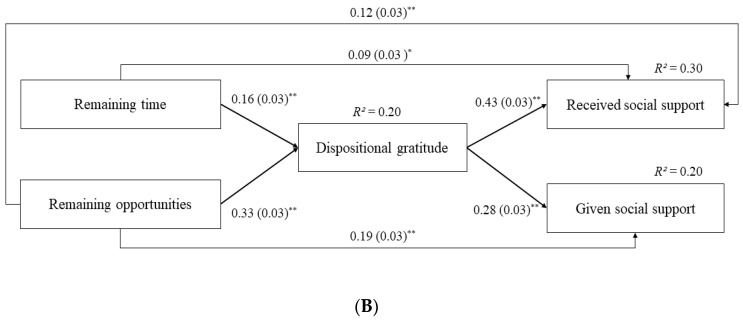
Mediation Models. (**A**) Original partial mediation model; (**B**) Alternative mediation model with no direct association of remaining time with given social support. Standardized path estimates are reported. Standard errors are in parentheses. Dotted lines indicate nonsignificant paths. The effects of gender and education attainment are omitted from the figures for clarity. * *p* < 0.01; ** *p* < 0.001.

The partial mediation model showed a good fit to the data (*χ^2^*(2) = 7.85, *p* = 0.02; RMSEA = 0.05; SRMR = 0.01; CFI = 1.00; TLI = 0.96), after controlling for gender and educational levels. Both dimensions of future time perspective were significantly, positively associated with the perception of greater received social support directly and indirectly, through higher dispositional gratitude. Regarding the association with given social support, it was direct and indirect, significant, and positive for remaining opportunities, but only indirect, significant, and positive for remaining time. Because the direct association of remaining time with given social support was nonsignificant, we tested an alternative model in which this association was dropped, and we compared it against the original partial mediation model. The alternative model (Figure 1; panel B) also fitted the data well (*χ^2^*(3) = 9.51, *p* = 0.02; RMSEA = 0.04; SRMR = 0.01; CFI = 1.00; TLI = 0.97). The Δ*χ^2^* test comparing the fit of the original and alternative mediation models was nonsignificant (Δ*χ^2^*(1) = 1.65, *p* = 0.20), indicating that the two models fitted the data equally well. Therefore, the more parsimonious model was preferred, in which gratitude completely mediated the association of remaining time with given social support. In the selected model, standardized path estimates for gender (coded 0 = women and 1 = men) were *b* = −0.16 (*SE* = 0.05), *p* = 0.001 on received social support, and *b* = −0.40 (*SE* = 0.05), *p* < 0.001 on given social support. Standardized path estimates for education (coded 0 = up to secondary school and 1 = up to postgraduate education) were *b* = 0.17 (*SE* = 0.05), *p* = 0.001 on dispositional gratitude, and *b* = 0.01 (*SE* = 0.04), *p* > 0.05 on received social support. Total and indirect effects are displayed in Table 3, where confidence intervals that do not include zero are considered significant. 

## 4. Discussion

The present study aimed to investigate whether future time perspectives, conceptualized as remaining time and opportunities, were associated with perceived social support received from and given to others, directly and indirectly, through the mediation of dispositional gratitude. Although gratitude was associated either with time perspective or social support [6,7,42], no study has simultaneously considered all three dimensions, also taking a multidimensional approach to both future time perspectives and perceived social support.

Altogether, the findings supported our initial hypotheses. The first hypothesis on the direct associations of future time perspective with perceived social support was supported for the positive direct relationships of remaining time and opportunities with received social support. Thus, the higher the disposition to perceive the future as expanded and rich of opportunities, the higher the extent to which individuals report receiving social support from others. This finding is in line with previous research indicating that highly future-oriented people report more socially supportive relationships [5,12,43]. This may be attributable to that individuals who experience their future time as expansive tend to prioritize social-acceptance goals such as having people who accept, support, and trust them, as initially reported by Lang and Carstensen [4] and then replicated on pan-European data [44]. Remaining opportunities were directly associated also with higher perceptions of social support given to others. This finding is in line with evidence that people striving for future goals and rewards are more likely to engage in prosocial behavior [45]. Coherent with our findings, two experimental studies on future-oriented thinking found that participants who performed a writing task on their future life plans reported higher intentions to help others than participants who wrote about their present daily routine [19] or about past general or prosocial activities [46]. Writing about future life plans may induce goal setting and pursuit, which is an inherent aspect of focusing on remaining opportunities [6,16].

Contrary to our hypotheses, remaining time was not directly related to giving social support. Thus, the perception of having an extensive amount of time ahead might not be directly relevant to providing social support to others. Although this finding deserves further investigation, it suggests that the two dimensions of future time perspective are uniquely associated with the social support given to others. This interpretation is supported by factor analytic findings indicating that remaining time and remaining opportunities are two nonoverlapping components of future time perception [6,10,47,48]. 

The second hypothesis on the indirect association of future time perspective with social support through dispositional gratitude was supported. However, contrary to expectations, dispositional gratitude acted as a complete mediator between remaining time and social support given to others. Perceiving one’s future as broad and filled with remaining opportunities was linked to a greater tendency to feel grateful, and such a grateful outlook was associated with higher perceptions of received and given social support. Altogether, these indirect associations are consistent with recent evidence of a positive relationship between the perception of remaining time and opportunities in the future and dispositional gratitude [6,7,10,21]. They are coherent with the relationship-building inherent nature of gratitude [49]. They also reinforce previous evidence that dispositional gratitude promotes positive relations with others [50] and has a positive influence on perceived social support as both received from and given to others [18,19,51,52,53,54]. However, this is the first study that put together the three constructs. 

Noteworthy, looking at the standardized beta-values, focus on remaining opportunities was more strongly related to dispositional gratitude than focus on remaining time, which was also reported by Allemand and Hill [6,16]. Moreover, gratitude was associated more strongly with received than with given social support, which is in line with previous findings of a strong relationship of gratitude with received support [24,53] and of a moderate one with pro-sociality [42,54]. 

Altogether, the present research provided correlational evidence that adds to current knowledge on the link between future orientation and social support. Those people who exhibit a positive attitude toward the future tend to be high in gratitude and are thus more likely to recognize social support from others and engage in supportive behavior. Replication and longitudinal studies are nonetheless needed to corroborate our results. Future research might also explore the mediating role of gratitude in the relationship between future time perspective and different outcome variables that previous research identified as influenced by the perception of time, such as indices of positive and negative well-being (e.g., life satisfaction, subjective health, anxiety, and depression), or health and risk-taking behaviors [1,55,56]. 

Several limitations are worth noting in the present study. First, the correlational design does not allow causal conclusions; therefore, longitudinal research is needed to prove the causal influence of future time perspective and gratitude on perceived social support and to rule out alternative explanations. Second, we did not ask participants for the specific sources of perceived social support (e.g., significant others, family, acquaintances), which might moderate the associations found with reports of social support [5] and is thus worthy of being considered in future research. Third, we used only self-reported instruments; thus, we cannot exclude that the associations between variables were inflated as they shared a common method of assessment. Future studies are recommended that integrate information from multiple informants and behavioral measures and use more objective assessments of the individual’s social support network. Finally, caution should be used to generalize our findings due to the convenience sampling strategy used.

Despite the correlational nature of this research, the results might have some practice implications. Future time perspective and dispositional gratitude both represent a resource for positive social functioning [3,4,5,6,7,57]. Although changing one’s time perspective is not easy, a balanced time perspective can be fostered with appropriate training [30,58], with potential benefits for the individuals’ tendency to feel gratitude [28]. A number of positive psychology interventions also exist that are effective in promoting a grateful mood or disposition [31,59]. Further longitudinal studies are needed to verify whether training people in developing a more positive future perspective and a higher level of gratitude can improve their perception of receiving and giving social support with a consequent, positive impact on their psychological health.

## 5. Conclusions

The current study is unique in that we investigated the conjoint effect of future time perspectives and dispositional gratitude in the explanation of perceived social support intended as both received and given. Our findings suggest that future time perceived as expansive and full of remaining opportunities may influence one’s subjective experience of more positive social exchanges and this passes through dispositional gratitude, which mediates this relationship. Thus, our research highlights the importance of considering dispositional characteristics, such as the tendency to feel gratitude, to understand the impact of the subjective representation of the future on individuals’ social experiences. 

## Figures and Tables

**Table 1:** Descriptive Statistics of Psychological Variables (*n* = 1256).

	Range	M	SD	1	2	3	4
1. FTPS-T	3–15	9.68	2.79		-		
2. FTPS-O	3–15	10.45	3.07	0.52			
3. GQ−6	6–42	29.29	6.30	0.33	0.42	-	
4. 2-Way SSS-Received	0–55	44.32	10.22	0.30	0.34	0.52	-
5. 2-Way SSS-Given	0–50	35.79	6.84	0.21	0.29	0.37	0.52

FTPS-T = Future Time Perspective Scale-Remaining Time; FTPS-O = Future Time Perspective Scale-Remaining Opportunities; GQ−6 = Gratitude Questionnaire−6; 2-Way SSS-Received = 2-Way Social Support Scale-Received; 2-Way SSS-Given = 2-Way Social Support Scale-Given. All correlation coefficients were significant at *p* ≤ 0.001.

**Table 2:** Differences in Gratitude and Social Support according to Gender and Educational Level.

	M	SD	M	SD	F(11254)	*p*	*d*
GQ−6							
Female vs. Male	29.41	6.45	29.15	6.10	0.51	0.48	0.04
Lower vs. Higher Education	28.50	6.26	30.16	6.22	22.21	<0.001	0.27
2-Way SSS-Received							
Female vs. Male	44.99	10.42	43.49	9.91	6.79	0.009	0.15
Lower vs. Higher Education	43.66	10.71	45.04	9.61	5.78	0.02	0.14
2-Way SSS-Given							
Female vs. Male	36.92	6.61	34.41	6.86	43.14	<0.001	0.21
Lower vs. Higher Education	35.58	7.17	36.03	6.45	1.38	0.24	0.07

*d* = Cohen’s *d*. GQ−6 = Gratitude Questionnaire−6; 2-Way SSS-Received = 2-Way Social Support Scale-Received; 2-Way SSS-Given = 2-Way Social Support Scale-Given.

**Table 3:** Total and Indirect Effects of the Selected Mediation Model.

Effect	*b*	*SE*	95% CI
Remaining time → Received social support			
Total effect	0.16	0.03	[0.09, 0.22]
Indirect effect	0.07	0.02	[0.04, 0.10]
Remaining opportunities → Received social support			
Total effect	0.27	0.04	[0.20, 0.34]
Indirect effect	0.14	0.02	[0.11, 0.18]
Remaining time → Given social support			
Indirect effect	0.05	0.01	[0.03, 0.07]
Remaining opportunities → Given social support			
Total effect	0.28	0.03	[0.23, 0.35]
Indirect effect	0.09	0.01	[0.07, 0.12]

*b* = standardized estimate; *SE* = standard error; CI = confidence interval.
